# Geospatial characterization of rural settlements and potential targets for revitalization by geoinformation technology

**DOI:** 10.1038/s41598-022-12294-2

**Published:** 2022-05-19

**Authors:** Yixuan Liu, Xinxin Ke, Weicheng Wu, Ming Zhang, Xiao Fu, Jie Li, Jingheng Jiang, Yecheng He, Cuimin Zhou, Wenjing Li, Yuan Li, Yifei Song, Xiaoting Zhou

**Affiliations:** Key Laboratory of Digital Lands and Resources and Faculty of Earth Sciences, East China University of Technology, Nanchang, 330013 Jiangxi China

**Keywords:** Environmental sciences, Environmental social sciences

## Abstract

To better implement the Strategy of Rural Revitalization, it is essential to characterize the rural settlements and understand their roles in the socio-environmental interactive system. This paper is hence aimed at achieving such a study using different spatial analysis such as kernel density and spatial autocorrelation (SA) and modeling approaches, e.g., simple and multiple linear regression analyses taking Jiangxi, a province in China as an example. Remote sensing, topographic and socioeconomic data were employed for this purpose. Through these analyses, it is found that the rural settlements in the study area appear to have a spatial distribution pattern of “dense north and sparse south” as an “F” type, and are quantitatively characterized as low elevations, flat terrain, high river and road densities, rich cultivated land resources and susceptible to the impact of urban radiation with a R^2^ of 0.520–0.748. Based on this understanding, a new inequality evaluation indicator of rural development, i.e., socio-environmental evaluation index (SEI), was developed. Areas with SEI lower than 0.40 should be given a priority to implement the revitalization strategy in the province. This index can also be extended to study of the imbalance of rural development in other regions and countries.

## Introduction

Rural settlements are the spatial carriers of rural people’s living, production, cultural inheritance, and other socioeconomic activity^1,2^. The settlements and their organizational pattern are not only one of the main research topics of rural geography, but also a key component of the man-nature interaction research, which is critical for understanding the rural structure, functional reorganization, optimization and their renewal^3^.

Rural settlements are not an occasional product of human activity. As “Hanshu (Book of Han Dynasty, AD 105)” depicted, “after a long peace without war and harmful disaster, local people started to build houses in the fringes of urban, and little by little, there appeared the villages”^4^. The scale, density and distribution of rural settlements are different from each other due to the different historical background, natural and socioeconomic conditions^4^. Some rural settlements still remain as villages even today since their occurrence but the others have been developed into big cities favored by geographical, political and socioeconomic conditions. Kusamitu considered that the spatial distribution pattern of the rural residential areas are result of a comprehensive action of the geographical elements^5^. However, the social and economic factors are also determinants of the distribution of rural settlements and their development, and these drivers promote not only change in society, production manner, and culture inheritance but also new policy making for rural areas^3,6–10^.

Rural research has been focused on the following aspects^11^, firstly, the relationship between rural settlements and socioeconomic factors, e.g., impact of villages on the layout of public service facilities, community governance, rural crime and security, rural poverty and improvement of rural income^12–17^; secondly, the spatial distribution pattern, evolution mechanism, protection and renewal of villages^18–23^; thirdly, development influencing factors or driving forces^1,2,24–28^, and at last, optimization of residential areas^29^. These studies, focused on the spatial distribution of traditional villages or small-scale rural settlements at local scale, e.g., at county level, have improved our understanding of village distribution patterns and their influencing factors in general, and the relation with social problems in rural areas but none of them has tackled the priority targeting for revitalization at regional level (e.g., provincial level). It is hence of importance to conduct such a research to sort out the technical issue to meet the need of implementing the revitalization strategy.

Actually, since 1949, labor force has been liberated and the rural areas have been developed to a certain extent^30^ but the major problem faced was the poverty in China before 2000. The governmental efforts in rural areas were thus focused on eradication of poverty through agricultural reform and improvement of the transport system in countrysides since 1978. Especially, after implementation of the “Reform and Opening” policy by Xiaoping Deng in 1987, market economy had become predominant and house-hold and township enterprises based on the rural collective economy had been developed rapidly^31^, and the construction of villages reached its peak in the late 1990s^32^. A great achievement has been reached in poverty alleviation, e.g., population in poverty dropped from 770 million (97.5% of the total population) in 1978 to 16.60 million in 2018^33^. However, despite some measures such as development of local-suited plantation, transport system, fabrication industry and the bilateral joint economy, etc., have been taken to revive rural areas while alleviating the poverty, a major trend arising is the emerging urbanization in the whole country since 1990s. Various factors of production such as land resources, funding possibility and technologies have been gradually concentrated in cities and their suburb areas, and a great number of rural settlements have become “hollowed out” with a stagnancy or even decline in population and economic development as young labor force has been migrating to and living in cities. For this reason, a new strategy on rural revitalization was promulgated by the Central Government in 2018^34^, and a number of policies of priority, such as improving the rural living environment, promoting the rural “toilet revolution”, and establishing a modern agricultural production system and improvement of income, etc., have been proposed since then. This raises an issue where and how this revitalization strategy should be implemented in rural areas.

With the advancement of the geographic information science (GIS) and remote sensing (RS) technology in recent decades, accurate and rapid characterization of the spatial distribution and development trends of rural settlements has been made possible^6,7,21,26,35–37^. Based on the above understanding, the main purpose of this research is to use geoinformation technology including GIS, remote sensing and spatial modeling to explore the actual situation of rural settlements and their relationship with social and environmental factors; at the same time, to formulate a new index to evaluate the development inequality of rural areas in order to provide target areas and relevant suggestions for the government to better implement the Rural Revitalization Strategy taking Jiangxi Province in China as an example.

## Material and methods

### Study area

**Figure 1:**
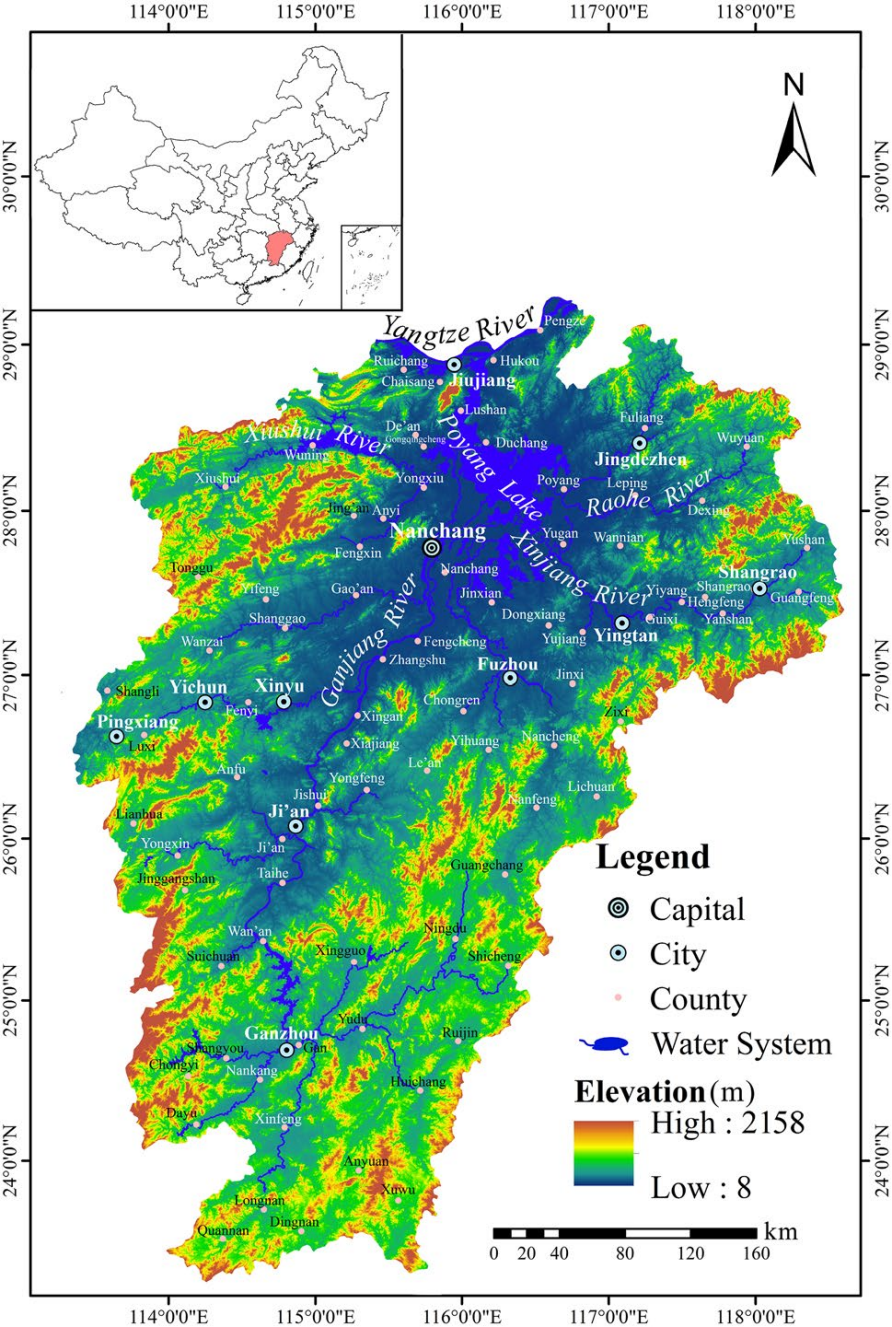
Location of the study area, Jiangxi Province in China and its landform characteristics. Note: Map was created by authors using ArcGIS 10.2 and Adobe Illustrator CS6 based on the digital elevation model (DEM) data from NASA (www.earthdata.nasa.gov).

Jiangxi is a province located in East China and encompasses the Poyang Lake Basin, a sub-tributary watershed on the south bank of the middle reaches of the Yangtze River. It is geographically situated between 113° 34′ 36″ E and 118° 28′ 58″ E in longitude and 24° 29′ 14″ N and 30° 04′ 41″ N in latitude, covering an area of 166,900 km^2^. Jiangxi belongs to the subtropical monsoon climate zone with four distinct seasons, humid and hot summer and cool winter. The annual average rainfall is about 1642 mm, mostly concentrated in March to July while the annual average temperature is 17.9 °C. The elevation of the study area varies from 8 to 2158 m above sea level (Fig. 1). The population was approximately 46.66 million in 2019 and the province has a jurisdiction over 11 prefecture-level cities, 27 municipal districts, and 73 counties.

### Data

This study employed very high resolution images (1–2.5 m) available on Google Earth to vectorize the rural settlements (villages) and roads dated Dec 2010, Dec 2015 and Dec 2019.

**Figure 2:**
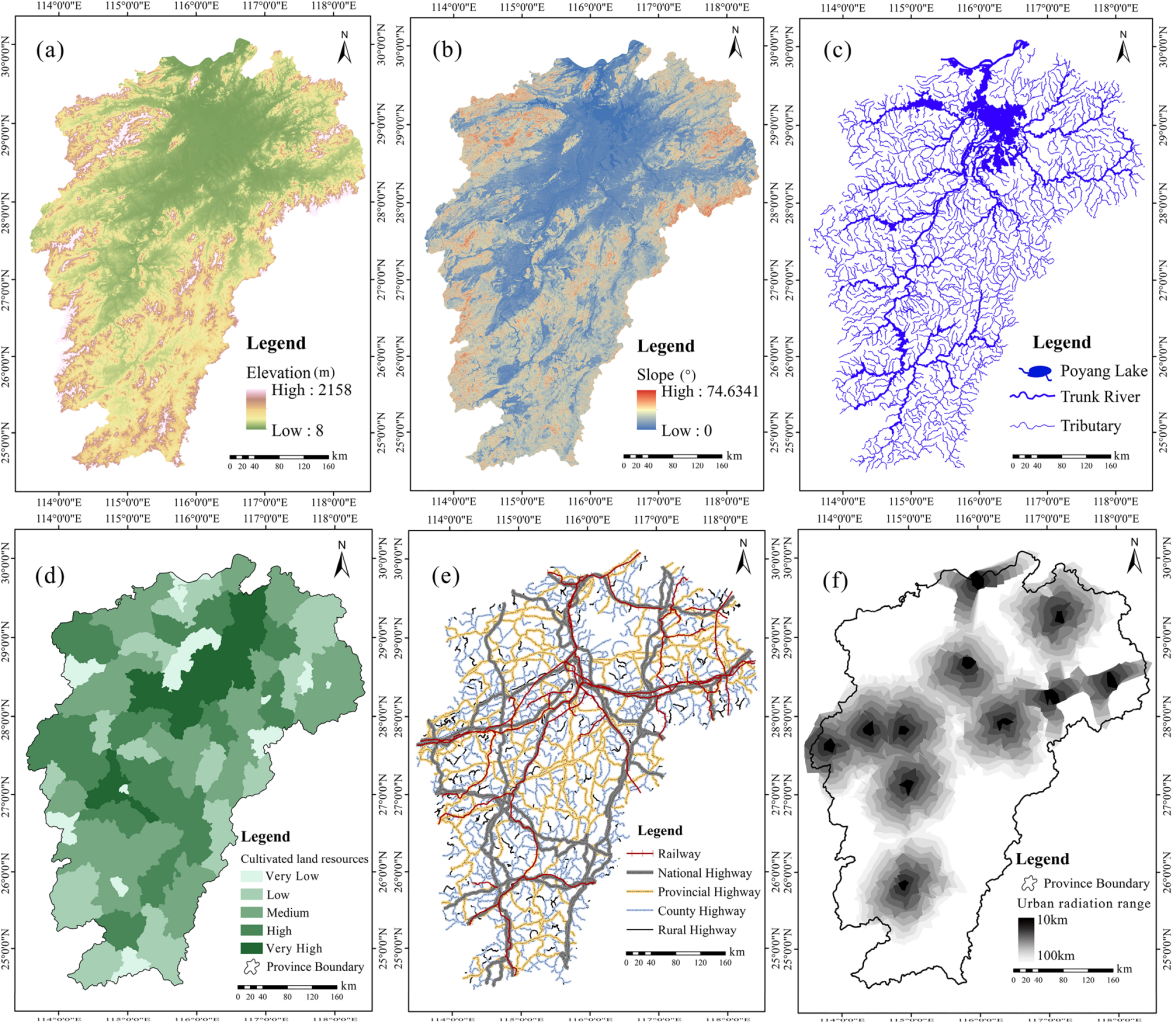
Factors influencing the distribution of the rural settlements: (**a**) elevation, (**b**) slope (**c**) river networks, (**d**) cultivated land resources, (**e**) road networks, (**f**) city-town radiation effect. Note: All these maps were created by authors using ArcGIS 10.2 but maps **a**, **b** and **c** were based on the digital elevation model (DEM) data from NASA (www.earthdata.nasa.gov); map **d** was derived from the land use data (30 m) of the Chinese Academy of Sciences (https://www.resdc.cn/); map **e** digitized from Google Earth (©Google ) and map **f** generated using network analysis within ArcGIS.

Digital elevation model (DEM) data (ASTGTMV003, 30 m) (Fig. 2a), obtained from NASA (www.earthdata.nasa.gov), were used for deriving environmental elements such as slope (Fig. 2b), aspect and rivers (Fig. 2c).

Socioeconomic data at county-level including rural population, GDP, per capita GDP, per capita rural income, cultivated land, were extracted from the 2010 and 2019 Statistic Yearbooks of Jiangxi^38^.

Land use and land cover maps (30 m) were obtained from the Institute of Geographical Sciences and Natural Resources Research (IGSNRR), Chinese Academy of Sciences (https://www.resdc.cn/) for the study area dated 2010, 2015 and 2019. They were quantified to county-level and that of 2019 is presented in Fig. 2d.

Field data related to distribution of a part of villages in the study area and their geographic environment and historical background were acquired during the field survey in Oct 2019, Aug 2020, Jul 2021 and Dec 2021. Field investigations were conducted with help of OvitalMap, a mobile phone App that allows to position accurately the observed locations with Global Positioning System (GPS) or Beidou System and record the investigation, e.g., geographical environment of the village, its scale, population and historical evolution by inquiring villagers and visiting their history museums.

Finally, through data collation and aggregation, a spatial database of rural settlements of Jiangxi was prepared.

### Methods and procedures

#### Vectorization of settlements and derivation of socio-environmental factors

##### Vectorization of settlements

Identification and vectorization of rural settlements were effectuated on Google Earth. It is worth mentioning that villages with less than about 9–10 houses were not vectorized because of difficulty to match the digital elevation model (DEM) data. Supposing that one house covers an area of about 10 m × 10 m = 100 m^2^, 10 houses is around 1000 m^2^, more or less similar to the pixel size of the DEM data,30 m × 30 m = 900 m^2^. Such a size is too small to have representative in spatial analysis when using DEM data. Another factor considered is that villages should have already existed before 1985. We noted from field surveys that recent extension or new villages development has been taking place along or around roads, revealing that many artificial interventions have played an active role in settlement extension and downplayed the roles of the natural environment in recent three decades. Thus, those unrecognizable in Landsat TM images dated 1984/1985 on Google Earth were not selected for this study. Hence, villages with more than 10 houses together with all commune-level towns in each county were digitized and finally 8,883 rural villages were obtained.

The prefecture-level cities and county-level towns, i.e., where the county-level governments are located, in total 92, were also vectorized.

##### Derivation of slope, aspect, rivers and watersheds

The DEM data were harnessed to derive slope in degree (Fig. 2b), aspect, rivers (Fig. 2c) and watersheds. River networks was extracted after depression filling, flow direction determination, and calculation of the concentrated flow accumulation and river length. An initial calculation shows that the minimum surface area of the big river watersheds is greater than 1000 km^2^, and the maximal area of the smallest rivers, or rather, the catchment of streams, is less than 100 km^2^. Thus, our idea was to set up the corresponding thresholds (pixel number) to divide three levels of watersheds, e.g., < 100 km^2^ (111,000 pixels) for Level 3 catchments, i.e., the valleys in mountains or hills where streams or small rivers develops; 100–1000 km^2^ (1.11 × 10^–6^ pixels) for Level 2 watersheds, or rather, the tributary watersheds of the main rivers; and > 1000 km^2^ for Level 1 watersheds, the five main big river basins, more concretely, where the major trunks are located. For simplification, we set 1,000,000 and 100,000 pixels as thresholds for this division. The three levels of rivers were extracted from their corresponding watersheds and then vectorized to constitute river networks (Fig. 2c), and they were further summed at county-level to calculate the river network density.

##### Road digitization and density calculation

Referring to the available maps such as BaiduMap (https://map.baidu.com/) and Amap (https://ditu.amap.com/), roads of different levels such as railways, highways, national highways, provincial roads, county-level roads, rural (town-level) roads were vectorized from Google Earth (Fig. 2e). Moreover, in terms of the Statistic Yearbooks of Jiangxi of 2010 and 2019^38^, using road length, road network density was calculated for each county.

All the above intermediary results were incorporated into the above geospatial dataset of the rural settlements of the study area.

##### Scope of city-town radiation

Based on the economic scale of the cities and accessibility to them by transportation, the scope of city-town radiation was visualized by network analysis and that of prefecture-level cities is shown in Fig. 2f.

#### Spatial analysis

Kernel density (KD), a non-parametric method to estimate the probability density of a random variable^39^, was used to analyze the spatial distribution pattern of the rural settlements by calculating the unit density within the specified neighborhood, and the result generates a smooth surface with a large median and a small peripheral values. It can be expressed as:
  1 $$f\left( {x,y} \right) = \frac{1}{{nh^{2} \mathop \sum \nolimits_{i = 1}^{n} K\left( {\frac{{d_{i} }}{h}} \right)}}$$
  where *f* (*x, y*) is the density estimation at the location (*x, y*), *n* the number of rural settlements, *h* the kernel size, *K* the size of land areas, i.e., the kernel function, and *d*_*i*_ the distance between the location of the *i*th rural settlement and the location (*x, y*)^36,40^.
  KD is an unbiased probability density estimation approach supported by complete mathematical theory and has good statistical performance. Among the commonly used non-parametric probability density estimation methods, the theoretical development of KD estimation appears to be the best^41^. Meanwhile, it is more suitable for analyzing the dimensional data related to population and those in geography than other estimation approaches^42–44^.
Spatial autocorrelation (SA) analysis is measure whether one observation in space is correlated with its neighbors^45,46^, including SA (Moran’s I), and cluster and outlier analysis (Anselin local Moran’s I).
  Global SA is a description of the spatial distribution characteristics of attributes in the entire region^19,47^, which is usually measured by Moran’s I to determine whether there are spatial clusters or outlier at global level. The equation for calculation is shown as follows:
  2 $${\text{Morans }}I = \left[ {n\left( {\mathop \sum \limits_{i = 1}^{n} \mathop \sum \limits_{j = 1}^{n} \omega_{i,j} z_{i} z_{j} } \right)} \right]/\left[ {\left( {\mathop \sum \limits_{i = 1}^{n} \mathop \sum \limits_{j = 1}^{n} \omega_{i,j} } \right)\mathop \sum \limits_{i = 1}^{n} z_{i}^{2} } \right]$$
  where *z*_*i*_ is the deviation of the attribute of element *i* from its average value (*x*_*i*_-$\overline{x}$), *ω*_*i, j*_ the spatial weight between elements *i* and *j*, *n* the total number of rural settlements. The value of Moran’s I is between [−1, 1]; when Moran’s I > 0, it means that the rural settlements are clustered or agglomerated in space; when Moran’s I < 0, the rural settlements are discrete in space; and when Moran’s I = 0, the rural settlements are distributed randomly.
  Cluster and outlier analysis is effectuated by Anselin Local Moran’s I. The formula is shown as follows:
  3 $$I = \frac{{x_{i} - \overline{X}}}{{S_{i}^{2} }}\left[ {\mathop \sum \limits_{j = 1,j \ne i}^{n} \omega_{i,j} \left( {x_{j} - \overline{X}} \right)} \right]$$
  in which
  4 $$S_{i}^{2} = \frac{{\mathop \sum \nolimits_{j = 1,j \ne i}^{n} \left( {x_{j} - \overline{X}} \right)^{2} }}{n - 1} - \overline{X}^{2}$$
  where *x*_*i*_ is the attribute value of element *i*, $\overline{X}$ the average value of the corresponding attribute, *ω*_*i, j*_ the spatial weight of element *i, j*, and *n* the total number of rural settlements.
(3) The nearest neighbor distance (NND) approach calculates the nearest neighbor index based on the average distance between each feature and its nearest neighbor^48,49^. The calculation formula is presented as follows:
  5 $$\overline{{r_{E} }} = \frac{1}{{2\sqrt {n/S} }} = \frac{1}{2\sqrt \omega }$$
  6 $$NNR = \overline{{r_{i} }} /\overline{{r_{E} }}$$
  where $\overline{{r}_{E}}$ is the theoretical NND, *n* the number of rural settlements, *S* the area of the study area, *ω* the point density, $\overline{{r}_{i}}$ the actual NND, and *NNR* the nearest neighbor ratio.
  According to the *NNR*, three types of distribution can be obtained: when *NNR* > 1, the actual nearest distance of the point element is greater than the theoretical nearest distance, that is, the rural settlements are uniformly distributed; when *NNR* = 1, they are distributed in a random pattern; and when *NNR* < 1, they are clustered. This method can be used for measuring the nearest neighbor ratio of the rural settlements, and thereby analyzing their spatial distribution patterns.

#### Regression modeling

Multiple linear regression models are able to reveal the most important factors in provoking the event, e.g., change or occurrence, which are regarded as dependent variable, and establish the intuitive models between the dependent and independent variables^7,50^. In our case, the coordinates of the settlements were considered as dependent variable and other socio-environmental factors as independent variables which were normalized using the Z-score approach (see variable names in Table 4. Importance of the socio-environmental factors in development of rural settlements resulted from simple linear regression analysis). Multiple linear regression modeling was conducted in a stepwise manner with least-square approach.

#### Socio-environmental evaluation index (SEI)

To explore where the Strategy of Rural Revitalization shall be first implemented in the studied province, we intended to develop a new SEI for this purpose. As village-level socioeconomic data are not available and we decided to utilize the county-level rural per capita income to represent the rural richness or poverty among the counties. Though Gini Coefficient^51^ and Engel’s Coefficient^52^ have been frequently applied to evaluate the inequality of development or poverty of countries worldwide, they use a single indicator such as income or percentage of income spent on food for this purpose, and thus have been criticized^53,54^. A simple correlation analysis uncovers that the rural per capita income at county-level is negatively correlated with elevation, slope and positively with cultivated land despite of a low significance (R^2^ ≦ 0.31) because of a homogenization of the elevation and slope data at county-level to match the rural income data. Hence, it came to our mind that it would be more reasonable to integrate the natural endowment into the rural per capita income for developing a new evaluation index, i.e., the Socio-environmental Evaluation Index (SEI), using the above mentioned indicators to evaluate the inequality of development in rural areas:

7 $$SEI = \frac{1}{{1 + e^{{ - \left( {X1*\left( {X2/\left( {X3*X4} \right)} \right)} \right)}} }}$$

where SEI is a logistic function and *X*_*1*_ is the county-level rural per capita income normalized using Z-score approach; *X*_*2*_ is cultivated land resource, *X*_3_ mean elevation and *X*_4_ mean slope at county-level respectively and are normalized by the max-min scaling approach. Taking slope *X*_4_ as an example, *X*_4_ = (*X*_*4i*_−*X*_*4min*_)/(*X*_*4max*_−*X*_*4min*_). The final SEI value comes between 0 and 1 similar to that of Gini Coefficient or Engel’s Coefficient, and the higher the SEI value, the less stressed the development of rural villages or vice versa.

## Results

### Spatial distribution pattern of rural settlements

**Figure 3:**
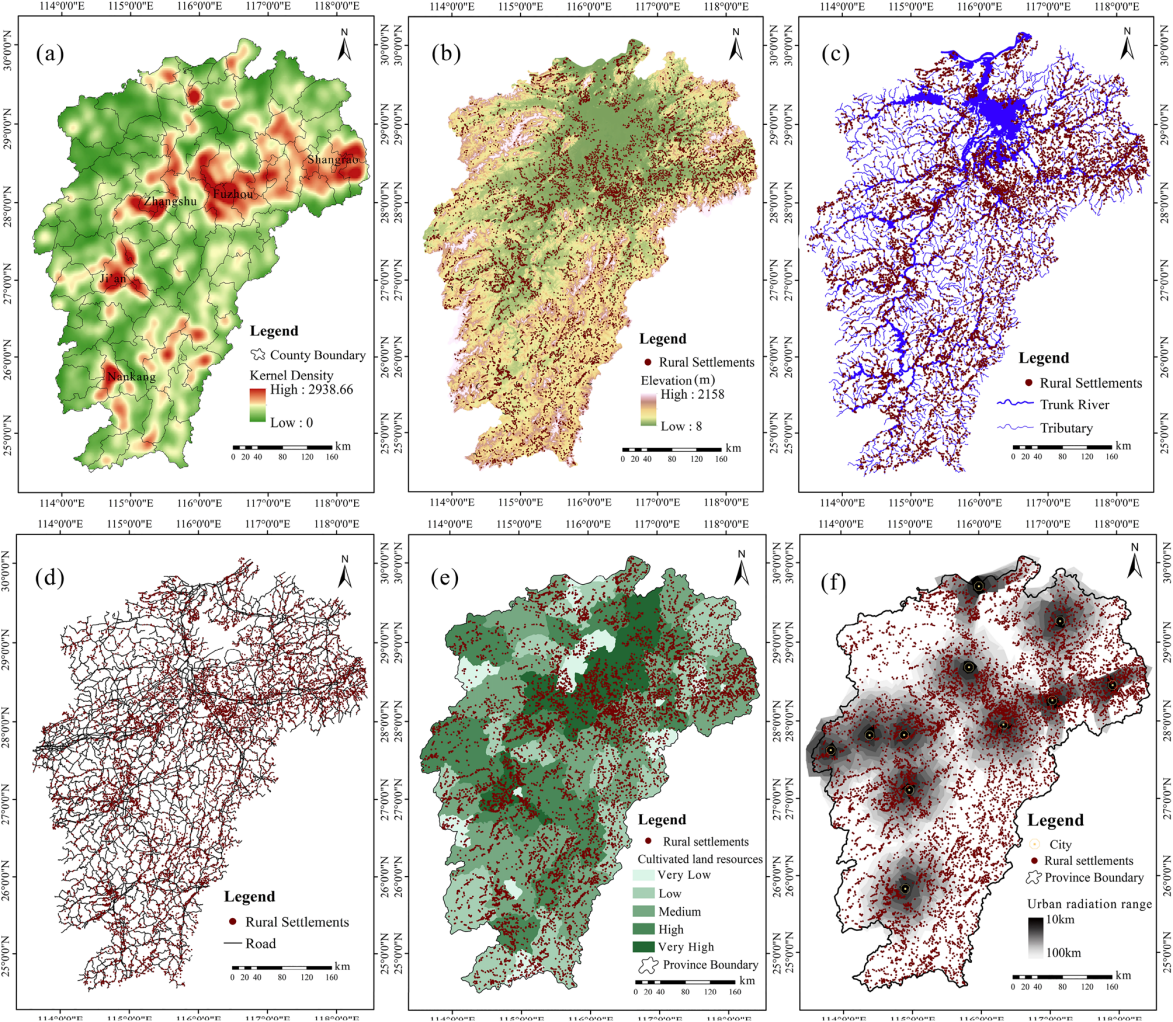
Spatial distribution of rural settlements of 2019 versus environmental factors: (**a**) the result of kernel density (KD) analysis, (**b**) villages and elevation, (**c**) villages and rivers, (**d**) villages and roads, (**e**) cultivated land resources, (**f**) city radiation effect. Note: All these maps were created by authors using ArcGIS 10.2.

There were 8883 rural settlements in the study area in 2019 and the overall spatial agglomeration characteristics and distribution map of the rural settlements are illustrated in Fig. 3.

As KD estimation revealed, the distribution of the rural settlements as a whole appears to be “dense north and sparse south” (Fig. 3a), which is similar to the population distribution in the province, and specifically, the rural settlements constitute a “F” type agglomeration in the study area.

*NNR* reveals that 82% of the rural settlements in 80% of the province’s jurisdiction area are in agglomeration state in which *NNR* lies in between 0.69 and 0.98, less than 1.0, indicating that most of the villages in the province have a high degree of agglomerated distribution.

### The spatial distribution and environmental factors

#### Topographic features

##### Elevation

**Figure 4:**
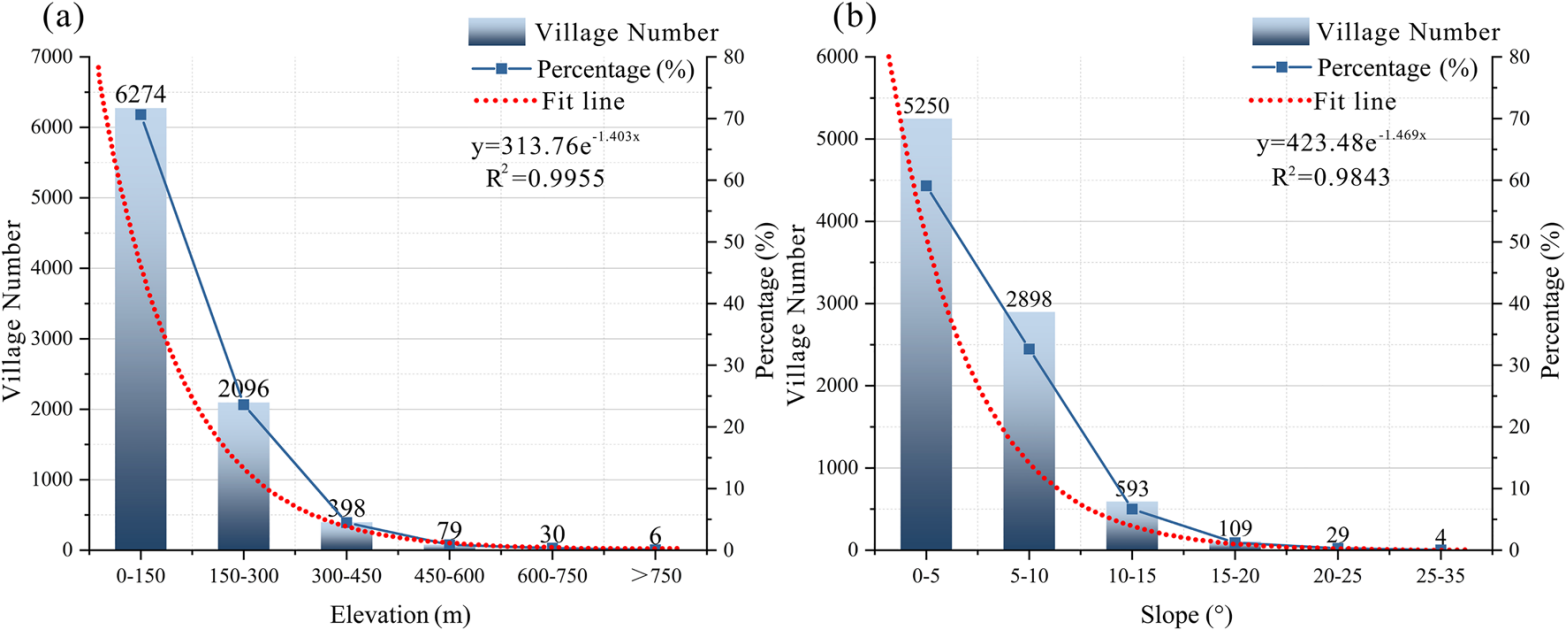
Rural settlements in different ranges of elevation (**a**) and slope (**b**).

The relationship between the elevation and spatial distribution of villages is presented in Table 1. Number of rural settlements in different ranges of slope, aspect and elevation in 2019 and Fig. 3b and Fig. 4a. Actually, there are 6274 (70.63%), 2096 (23.60%), 398 (4.48%), 79 (0.88%), 30 (0.33%) and 6 (0.06%) villages distributed in the altitude of 10–150 m, 150–300 m, 300–450 m, 450–600 m, 600–750 m and above 750 m respectively. That is to say, more than 93% of the rural settlements are located in plains or hilly areas below 300 m above sea level. Correlation analysis uncovers that the spatial distribution of villages is negatively correlated with elevation (Table 1. Number of rural settlements in different ranges of slope, aspect and elevation in 2019 and Fig. 4a), indicating that the lower the elevation and the flatter the terrain, the higher the suitability for village development. The Moran’s I is 0.638, demonstrating that the elevation and villages are spatially related and the latter is distributed in clusters.

**Table 1:** Number of rural settlements in different ranges of slope, aspect and elevation in 2019.

Slope (°)	Number	Percentage (%)	Area (km^2^)	Percentage (%)
< 5	5250	59.10	1533.71	62.62
5–10	2898	32.62	713.80	29.15
10–15	593	6.67	145.05	5.92
15–20	109	1.22	36.62	1.49
20–25	29	0.32	12.58	0.51
> 25	4	0.04	7.30	0.30

Aspect (°)	Number	Percentage (%)	Area (km^2^)	Percentage (%)
0–22.5, 337.5–360 (N)	1066	12.00	329.56	13.46
22.5–67.5 (NE)	1053	11.85	288.86	11.79
67.5–112.5 (E)	988	11.12	258.91	10.57
112.5–157.5 (ES)	1148	12.92	307.32	12.55
157.5–202.5 (S)	1387	15.61	368.83	15.06
202.5–247.5 (SW)	1223	13.76	317.67	12.97
247.5–292.5 (W)	1013	11.40	269.41	11.00
292.5–337.5 (WN)	998	11.23	296.51	12.11

Elevation (m)	Number	Percentage (%)	Area (km^2^)	Percentage (%)
10–150	6274	70.63	2022.92	82.59
150–300	2096	23.60	373.66	15.26
300–450	398	4.48	42.88	1.75
450–600	79	0.89	7.11	0.29
600–750	30	0.34	1.67	0.07
> 750	6	0.06	1.17	0.05

##### Slope and aspect

Statistical analysis revealed that the number of villages located in areas with a slope of 0°–5°, 5°–10°, 10°–15°, 15°–20°, and > 20° is respectively 5250 (59%), 2898 (32%), 593 (6.6%), 109 (1.2%), and 33 (0.3%). Clearly, more than 90% of the villages are distributed in areas with a slope below 10° (Table 1. Number of rural settlements in different ranges of slope, aspect and elevation in 2019 and Fig. 4b). There is an obvious negative correlation between the spatial distribution of the rural settlements and slope (Fig. 4b), implying that the smaller the slope, the better the clustering of villages.

Regarding the aspect, we noted that the rural settlements are more or less evenly distributed on all aspects in the study area and no aspect dominance is observed (Table 1. Number of rural settlements in different ranges of slope, aspect and elevation in 2019).

#### Rivers

As shown in Fig. 3c, Table 2. Number of rural settlements and river buffer zone in 2019 and Table 4. Importance of the socio-environmental factors in development of rural settlements resulted from simple linear regression analysis, the rural settlements are closely associated with rivers, in particular, with the tributaries of the main rivers (R^2^ = 0.748). Local autocorrelation analysis of rivers reveals that Moran’s I is 0.865, implying that river density has an evident impact on the agglomeration of rural settlements in space.

**Table 2:** Number of rural settlements and river buffer zone in 2019.

Buffers (m)	Main Rivers		Tributaries		Total	
	Number	Percentage (%)	Number	Percentage (%)	Number	Percentage (%)
1–100	0	0	513	9.25	513	8.82
100–200	4	0.99	900	16.24	905	15.57
200–300	20	4.93	803	14.49	824	14.18
300–400	35	8.62	680	12.27	718	12.35
400–500	49	12.07	459	8.28	508	8.74
500–600	38	9.36	424	7.65	452	7.78
600–700	35	8.62	283	5.11	312	5.37
700–800	40	9.85	265	4.78	300	5.16
800–900	36	8.87	201	3.63	225	3.87
900–1000	27	6.65	202	3.64	215	3.70
1000–1100	30	7.39	187	3.37	198	3.41
1100–1200	24	5.91	148	2.67	151	2.60
1200–1300	30	7.39	182	3.28	192	3.30
1300–1400	23	5.67	151	2.72	158	2.72
1400–1500	15	3.69	145	2.62	141	2.43

Moreover, buffering analysis finds out that there are 406 villages in the trunk river buffer zone, accounting for 4.57% of the total rural settlements, whereas there are 5,543 villages in the buffer zones of the tributaries, taking up 62.4% (Table 2. Number of rural settlements and river buffer zone in 2019). Actually, settlements are more concentrated in the zone of 500 m from the main trunks while they are distributed in the belt about 200 m from the tributaries on average. Hence, the tributaries have a greater impact on the distribution of villages than the trunks. With the increase of distance from the rivers, the number of villages gradually decreases. If the trunks and their tributaries were taken into account simultaneously, 5812 villages, 65.43% of the total, were found in the buffer zone of 200 m from the river tributaries.

#### Roads

**Figure 5:**
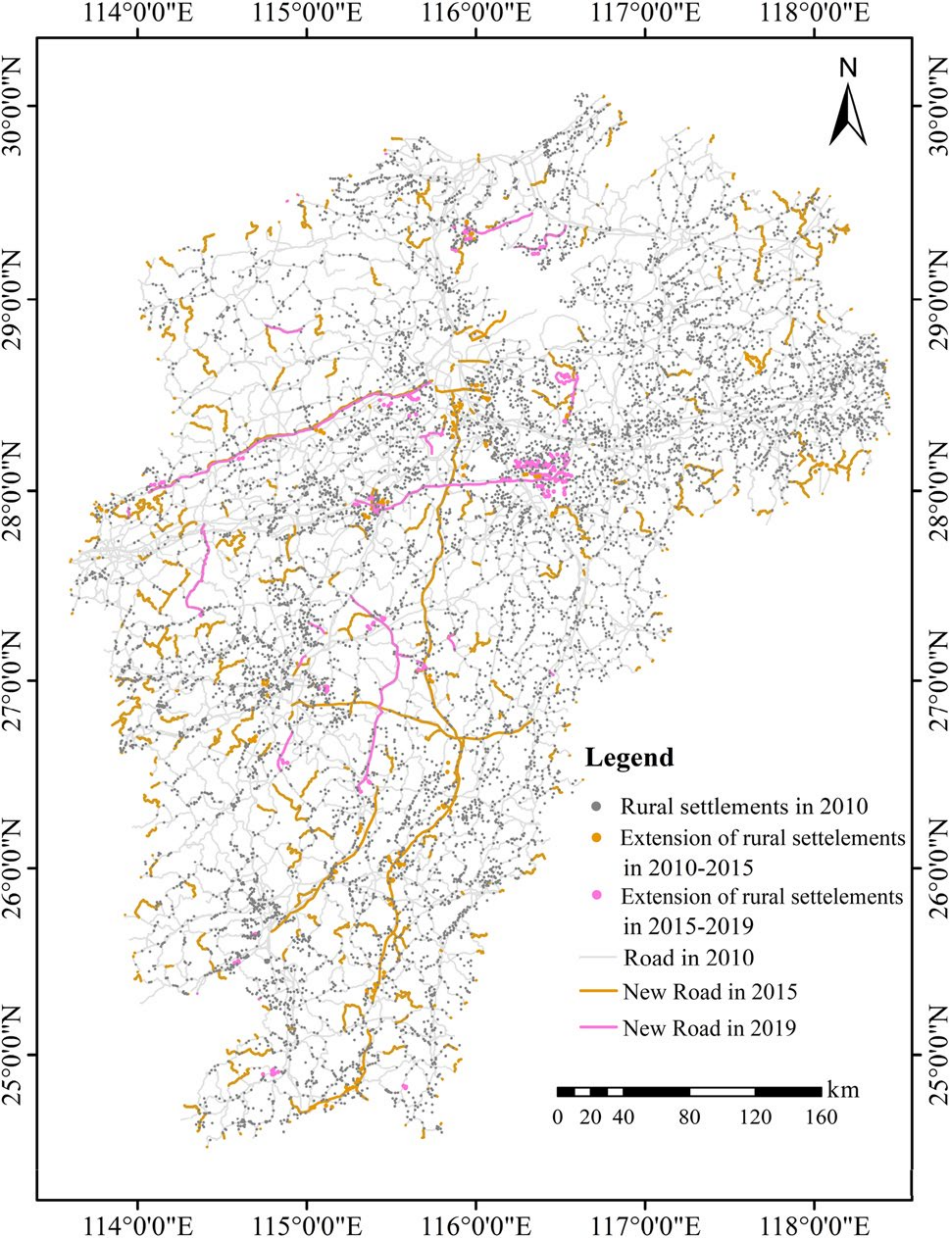
Extension of rural settlements along the road systems. Note: Map was created by authors using ArcGIS 10.2 based on digitization on Google Earth (©Google).

As a key spatial carrier of socioeconomic development, road system cannot only strengthen the connection between villages and cities serving for logistics and transport but also facilitate the development of rural settlements on the two sides of roads and, especially, at the nodes of the transport systems^55^. In this sense, roads exert an influence on spatial distribution pattern of the rural settlements. The road density at city-level represents the degree of regional transportation convenience or accessibility. The relationship between the spatial distribution of the rural settlements and the road system in the study area is shown in Fig. 3d. It can be intuitively seen that the more concentration the rural settlements, the denser the roads. This illustrates that the areas with higher accessibility are more conducive to agglomeration and development of villages. Actually, roads and rural settlements are the products of human socioeconomic construction, and they influence each other. In the recent decade, it is observed that most of the new extension of the rural settlements has taken places along the roads or at the intersected nodes of roads (Fig. 5).

The Moran’s I index of road density is 0.868, illustrating that the density of road facilitates the agglomeration of settlements in space.

Considering that the driving effect of road radiation has a certain range of limit, and the influence of different grades of roads on rural residential areas is also different, the buffer zones were constructed for different grades of roads, i.e., 150–1500 m multi-ring buffers for railway, 100–1000 m buffers for national highway, 80–800 m buffers for the provincial highway, 50–500 m buffers for county-level roads, and 30–300 m multi-ring buffer zones for the township-level roads (Table 3. Number of rural settlements within road buffers in 2019). The result of analysis shows that the county-level roads have the greatest impact on the development of the rural residential areas.

**Table 3:** Number of rural settlements within road buffers in 2019.

Railway buffers (m)	Number	National highway buffers (m)	Number	Provincial highway buffers (m)	Number	County-level road buffers (m)	Number	Township road buffers(m)	Number
0–150	71	0–100	109	0–80	209	0–50	315	0–30	16
150–300	147	100–200	187	80–160	316	50–100	517	30–60	29
300–450	103	200–300	128	160–240	212	100–150	419	60–90	26
450–600	90	300–400	114	240–320	149	150–200	276	90–120	26
600–750	89	400–500	112	320–400	136	200–250	196	120–150	27
750–900	80	500–600	81	400–480	101	250–300	154	150–180	16
900–1050	77	600–700	78	480–560	92	300–350	137	180–210	11
1050–1200	81	700–800	89	560–640	78	350–400	120	210–240	15
1200–1350	88	800–900	76	640–720	87	400–450	117	240–270	10
1350–1500	74	900–1000	77	720–800	75	450–500	93	270–300	10

#### Cultivated land resources

**Figure 6:**
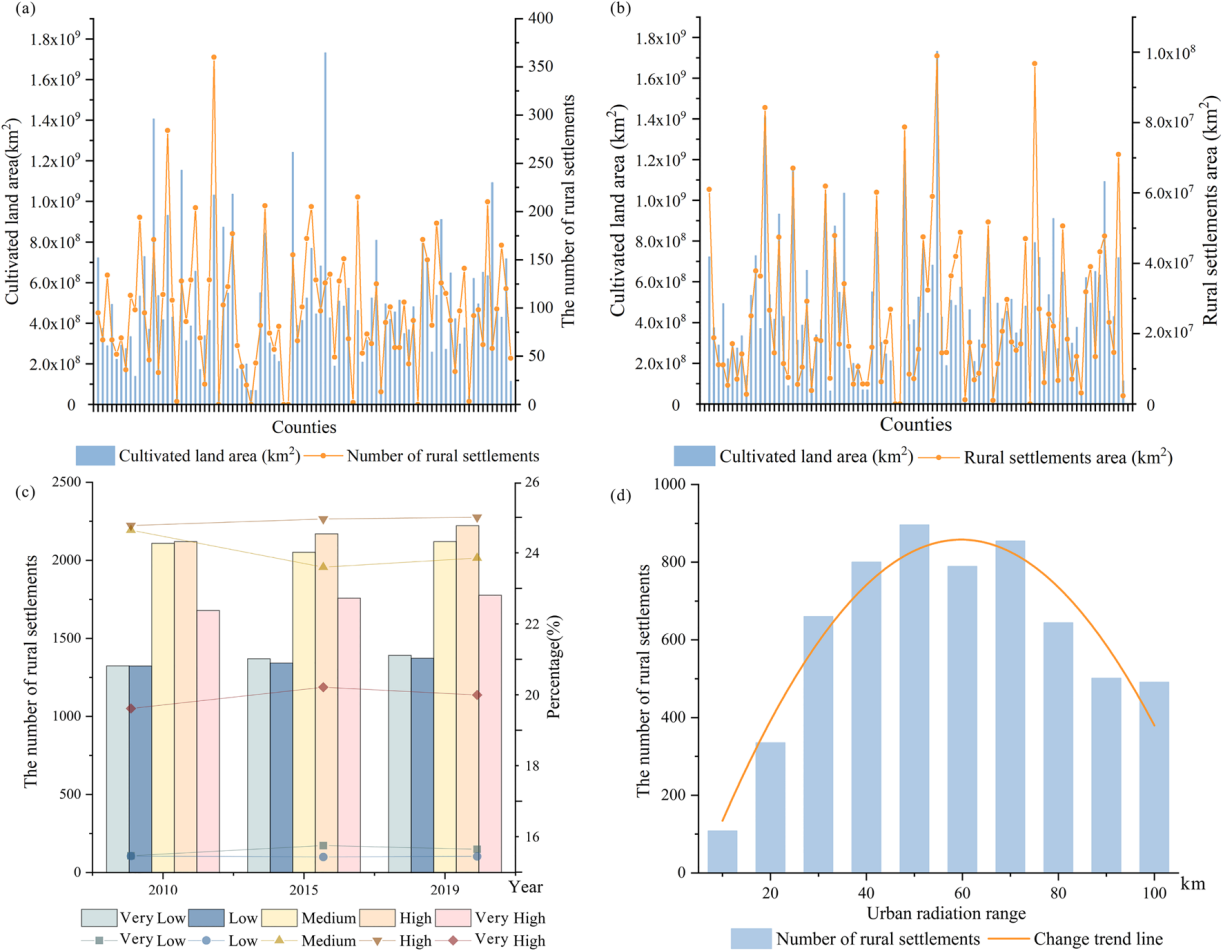
Relationship between the rural settlements and cultivated land resources and city radiation effect: (**a**) number of rural settlements in different cultivated land resources, (**b**) surface area of rural settlements in different cultivated land resources, (**c**) number of rural settlements in different cultivated land, (**d**) number of rural settlements in different urban radiation range.

As shown in Figs. 3e, 6a,b, and Table 4. Importance of the socio-environmental factors in development of rural settlements resulted from simple linear regression analysisand Table 5. Multiple linear regression model, cultivated land area and the number and area of rural settlements are positively correlated (R^2^ = 0.702). In areas with large cultivated land area, the number and area of rural settlements are large. This was confirmed by regression analysis, implying that the areas with rich cultivated land resources have a greater impact on development and spatial layout of rural villages with a higher density. Taking the past 10 years as an example (Fig. 6c), we noted that rural settlements have been greatly expanded in areas with rich and relatively abundant land resources or vice versa. In the period 2010–2019, 328 villages had experienced a significant extension either along the roads or in the cultivated land.

**Table 4:** Importance of the socio-environmental factors in development of rural settlements resulted from simple linear regression analysis.

Independent variable	Constant	R	F	*P*-value
Elevation (*x*_*1*_)	32.589	− 0.723	985.646	0.00
Slope (*x*_*2*_)	730.724	− 0.721	37.828	0.00
Aspect (*x*_*3*_)	4.726	0.010	0.539	0.02
Distance to main river (*x*_*4*_)	22.867	− 0.177	0.418	0.00
Distance to tributary (*x*_*5*_)	759.390	− 0.865	38.781	0.00
Distance to national highway (*x*_*6*_)	11.667	− 0.699	7.646	0.02
Distance to provincial highway (*x*_*7*_)	22.800	− 0.674	6.651	0.03
Distance to county-level road (*x*_*8*_)	37.800	− 0.770	11.661	0.00
Distance to town-level road (*x*_*9*_)	6.133	− 0.426	1.774	0.02
River network density (*x*_*10*_)	140.895	0.265	6.132	0.01
Road network density (*x*_*11*_)	130.628	0.248	5.315	0.02
Cultivated land area (*x*_*12*_)	27.615	0.838	65.504	0.00
Accessibility to cultivated land (*x*_*13*_)	25.511	− 0.148	0.075	0.00
Radiation of prefecture-level city (*x*_*14*_)	11.682	0.821	3.519	0.00
Radiation of county-level city (*x*_*15*_)	14.621	0.720	2.445	0.00

**Table 5:** Multiple linear regression model.

Independent variables	β	Sig	VIF
Elevation (*x*_*1*_)	− 0.587	0	1.755
Slope (*x*_*2*_)	− 0.079	0	1.044
Distance to main river (*x*_*4*_)	− 0.494	0	1.914
Distance to tributary (*x*_*5*_)	− 0.079	0	1.036
Distance to national highway (*x*_*6*_)	− 0.200	0	1.007
Distance to provincial highway (*x*_*7*_)	− 0.055	0	1.003
Distance to county-level road (*x*_*8*_)	− 0.291	0	1.000
Distance to town-level road (*x*_*9*_)	− 0.021	0	1.000
River network density (*x*_*10*_)	− 0.341	0	3.141
Road network density (*x*_*11*_)	0.358	0	3.018
Cultivated land area (*x*_*12*_)	0.153	0	1.049
Radiation of prefecture-level city (*x*_*14*_)	0.098	0	1.011
Radiation of county-level city (*x*_*15*_)	0.034	0	1.948
Constant	27.615	0	

The dependent variable is the spatial location of rural settlements and the independent variables in the table are socio-environmental factors.

The global SA Moran's I is 0.8173, exhibiting the linkage between the agglomeration of rural settlements and the cultivated land resources in space.

#### Radiation effect of cities

In line with urban population, cities are divided into different levels, which provide different services and impact scope (e.g., radii), influencing the spatial distribution of the rural settlements. Using network analysis, we obtained the service impact radii of cities and counties, and found that with the increase of distance, the number of rural settlements showed an “anti-U” trend in which it first increases and then decreases with the distance (Figs. 3f, 6d). Rural residential areas are mainly concentrated in an extent of 40–50 km away from prefecture-level cities and 9 km away from county-level towns. It is noted that for villages, the radiation effect of prefecture-level cities is much greater than that of county-level towns. 67.90% of villages are influenced by prefecture-level cities rather than by county-level towns, and only 5.25% of villages are more impacted by county-level towns than by prefecture-level cities.

### Factors impacting the spatial distribution of rural settlements

The results of simple and multiple linear regression modeling are listed in Table 4. Importance of the socio-environmental factors in development of rural settlements resulted from simple linear regression analysis and Table 5. Multiple linear regression model , depicting the relationship between the spatial distribution of rural settlements as dependent variable and the socio-environmental factors as independent variables. Through the P-P diagram test^56^, the results conform to a normal distribution. Their correlation with and combined action on the rural settlements are shown in Table 4. Importance of the socio-environmental factors in development of rural settlements resulted from simple linear regression analysis and Table 5. Multiple linear regression model. Clearly, the spatial distribution of the rural settlements is negatively correlated with elevation, slope, distance to river, distance to road and river network density. Among them, the negative correlation with the tributaries of rivers is stronger than that with the main rivers, and the county-level roads are more correlated with the rural residential areas than other level roads. Hence, it appears evident that the rural residential areas are more likely to agglomerate in areas with low slope, flat terrain, high river and road network densities, rich cultivated land resources and within the scope of urban radiation, as they provide favorable condition for settlement development. The developed multiple linear regression model is presented as follows:

8 $$\begin{aligned} & Y = - 0.587x_{1} - 0.079x_{2} - 0.494x_{4} - 0.079x_{5} - 0.200x_{6} - 0.055x_{7} - 0.291x_{8} \\ & \quad - 0.341x_{10} + \, 0.358x_{11} + \, 0.153x_{12} + \, 0.098x_{14} + \, 0.034x_{15} + 27.615 \\ \end{aligned}$$

where (*Y*) is the spatial location of rural settlements and *x*_*i*_ (*i* = 1, 2, 3, …. 15) is the socio-environmental factors as shown in Table 5. Multiple linear regression model . The adjusted R^2^ is 0.541, implying the reliability of the model. It seems reasonable that the distance from tributaries (i.e., proximity to river and water resources) and cultivated land resources (i.e., availability of food) among all the socio-environmental factors are the most important ones in the course of development of rural settlements, followed by radiation of the prefecture-level cities, county-level road and elevation, slope, etc., and aspect has the least importance.

### Target areas for implementing the Strategy of Rural Revitalization

**Figure 7:**
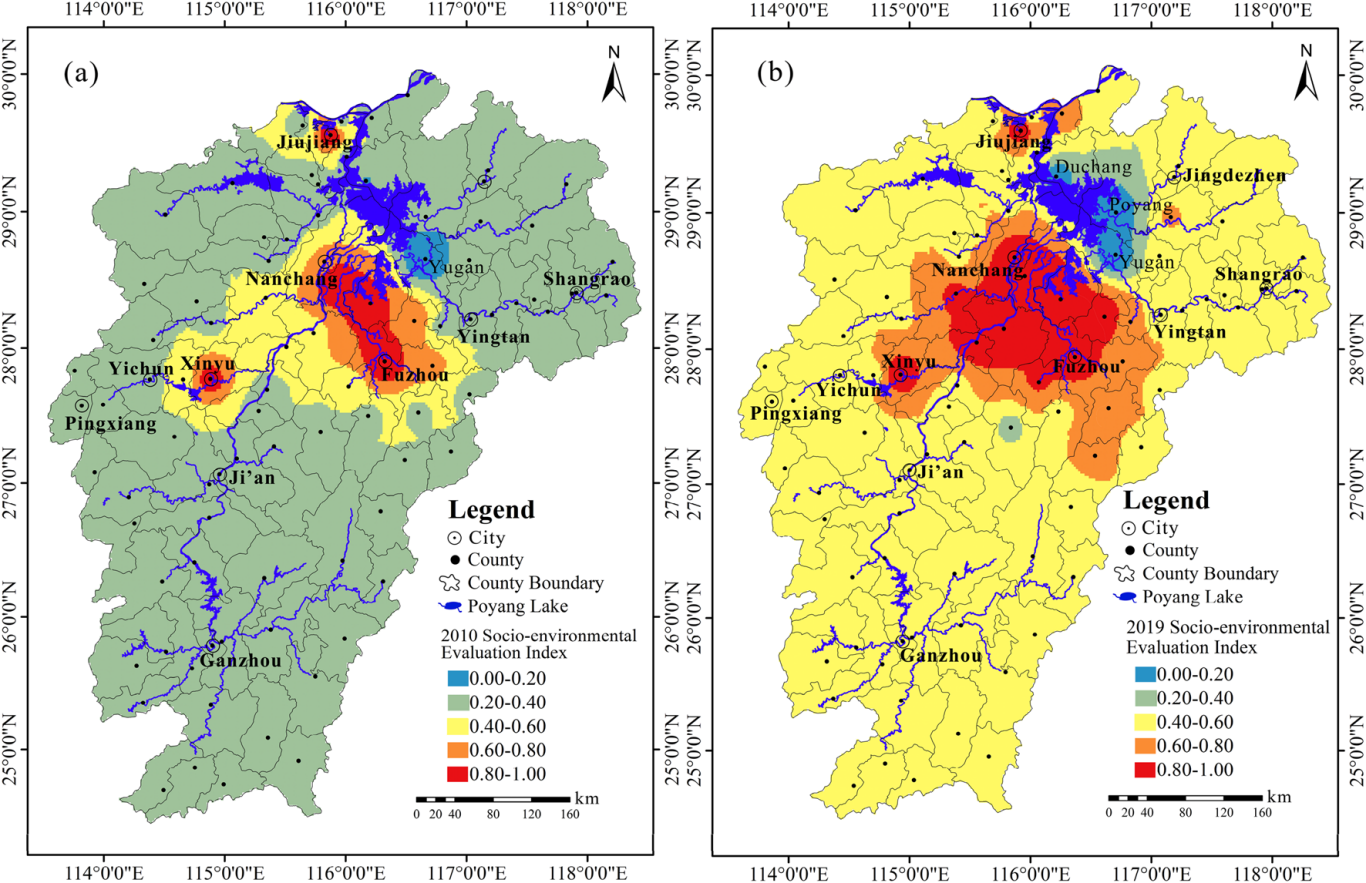
Inequality of rural development: Target areas of priority for implementing the Strategy of Rural Revitalization in Jiangxi. Note: Maps were created by authors using ArcGIS 10.2 based on the calculation of the Socio-environmental Evaluation Index (SEI) using county-level rural per capita income, county-level cultivated land area, mean elevation and mean slope: Maps of 2010 (**a**) and 2019 (**b**). Indication of the SEI grades: 0.00–0.20 Extremely stressed (Least developed) area, 0.20–0.40 Stressed area, 0.40–0.60 Moderate stressed area, 0.60–0.80 Low stressed area, and 0.80–1.00 Extremely low stressed (Most developed) area.

Resulted from Eq. (7), the rural development inequality maps of 2010 and 2019 are presented in Fig. 7. It is clearly seen that the rural areas of the whole province have developed into a new step in the past 10 years and the least stressed (or most developed) central area around Nanchang-Fuzhou cities has gained a significant extension. However, the inequality of rural development seems also enlarged between the central developed rural area and the eastern Poyang Lake area, notably, the counties Yugan, Poyang and Duchang, which are classified as stressed and extremely stressed area, and will be proposed as the first target area of priority for implementing the revitalization strategy.

## Discussion

### Influence mechanism of socio-environmental factors

In the process of village development, terrain, rivers, roads, cultivated land resources and city-town radiation are the fundamental factors influencing and determining the shape, scale and even the distribution pattern of settlements and their functions in society.

Located in the Poyang Lake Basin in the south of the middle reaches of the Yangtze River, the study area has been profiting the favorable natural conditions for rural settlement development as well as for agricultural production and transportation. Largely speaking, as the slope increases, the area suitable for cultivation decreases, and the cost for agricultural production increases. Therefore, the settlement development has been taking places in low altitude flat terrain with a proximity to rivers and roads, especially, to the tributaries and streams of rivers and to the county-level roads. As mentioned above, the formation of the “F” type agglomerations of rural settlements in the province (Fig. 3a) might have been influenced by the combined actions of rivers Ganjiang, Fuhe and Xinjiang, the radiation of the east–west Zhe-Gan (Zhejiang-Jiangxi) railway since 1929 and the south-north Beijing-Kowloon (Hong Kong) railway since 1996 as well as historical reason. It is obvious that the east–west conglomerated settlements appear to be much denser and larger than the south-north ones because the Zhe-Gan railway has much longer impact time than Beijing-Kowloon railway.

1. Elevation, slope and aspect: As depicted above, the lower the altitude and the slope, the more the advantage for settlement development. This is because of the topographic limitation as increase in altitude and slope will lead to an increase in difficulty for displacement, transportation, and utilization of water resources and a reduction in arable land resources.
  During the village formation, aspect is a complementary factor. Due to the difference in solar radiation, the sunny slope is warmer and direr than the shady slope, and is theoretically more suitable for crop production and habitation^36^. However, our analysis does not show any aspect preference of village development.
2. Rivers, tributaries and streams: It is commonly known that rivers play a critical role in the process of formation and development of the rural settlements as rivers provide vital water resources for life and agricultural production^57^, and at the same time, big rivers may serve for transportation of goods facilitating life and socioeconomic development, especially, in early time. Here are some examples, Meipo village in Ji’an by Fushui River and Jujing village in Shangrao surrounded by Xing River were first set up in Dynasty Song (960-1279AD), and Liukeng village in Fuzhou took its first place near Wujiang River in South Tang (937-943AD), etc. These old villages were all built along rivers or streams using the latter for water resources, irrigation as well as protection barrier. This shows the importance of rivers for development of rural settlements since history, and the influence of tributaries and streams is greater than that of the trunk rivers.. Thus, we may understand why people like to set up villages and towns near the rivers and their tributaries or around the harbors.
  Regression analysis revealed that village location is strongly and negatively correlated with rivers, especially, with the tributaries but the influence of rivers will decline with the increase in distance to tributaries or streams (Table 4. Importance of the socio-environmental factors in development of rural settlements resulted from simple linear regression analysis and Table 5. Multiple linear regression model). Furthermore, we found that the rural settlements are mostly concentrated in the range of 500 m away from the main river and 200 m away from the tributaries. This is to certain extent similar to the result of Qiu^37^. The main reason of such distribution is probably that the distance from the river will directly affect the production and life of residents. It is clearly more convenient for people to get water if nearer to the river, but settlements may face higher risk to flood disasters. Hence, it is essential to take both accessibility and security into account while developing settlements by rivers.
3. Roads: We also noted that roads are a decisive factor for agglomeration of settlements. At the beginning of village formation, rural roads did not form a systematic network but with the development of society and economy, road systems have been gradually improved. The convenience of transportation facilitates the renewal of the old settlements and also attracts construction of new settlements around and along the roads. This is confirmed by field observations and image analysis on villages and roads in recent ten years, e.g., village extension along the new roads constructed in 2010-2015 and 2015-2019 (Fig. 5). Our study reveals that the county-level road has a greater impact on the rural settlements than other level roads, which is similar to the conclusion of Chen^36^, but different from that of Yang^2^.
4. Cultivated land resources: As agricultural production resource, cultivated land plays a key role in socioeconomic development and is a critical nexus to understand the rural man-nature interactive relationship. Rich cultivated land resources are an attractive factor arousing people to build houses to settle down and thus lead to the development of the rural settlements as they are able to ensure the food security for people. That is why the village development is positively correlated with the cultivated land resources. This finding is supported by that of Yuan^58^.
5. Urban radiation effect: With urbanization, the impacts of prefecture-level and county-level cities on the development of rural settlements have become more and more evident. Cities are the central areas where commerce, education, health care, public services and other geographic functions are gathered and these constitute an socioeconomic factor impacting the development of villages. The closer to the cities, the stronger the effect to rural development. Compared with county-level cities, prefecture-level ones have a wider variety and range of services and opportunities, and hence play a more important role in development of rural settlements or extension of the old settlements. This result is similar to the one of Li^59^.

### Comparability

This research finds that the rural settlements in the study area as a whole show a spatial distribution pattern of agglomeration, which is comparable with the conclusions obtained from other regions in China. Pan depicted that the spatial distribution of rural settlements in the Loess Plateau lightly agglomerated^24^. The research of Xu also illustrated an obvious spatial agglomeration of rural villages in the oasis of Pishan, Xinjiang^60^, and Lim found that most of the villages in Cheongsan-do Island are concentrated in the valley plain in South Korea^61^.

Our analysis demonstrates a spatial directivity of roads and rivers in rural residential areas and their negative relationship (Table 4. Importance of the socio-environmental factors in development of rural settlements resulted from simple linear regression analysis and Table 5. Multiple linear regression model) and some socioeconomic factors such as cultivated land resources and urban radiation range will also exert an influence on the spatial distribution of rural settlements. This confirms the conclusion of Zhou i.e., there is a high spatial correlation between the cultivated land resources and the rural residential areas^62^.

However, some difference was also noted, e.g., our study did not find evident aspect of priority for settlement development in Jiangxi while Chen reported that a dominant aspect comes between 135° and 207° (from southeast to southwest) in Baota District in the Loess Plateau^36^. The reason of difference may lie in the fact that there is more precipitation and cloud in our study area, and the rainy weather might have made the aspect distribution unimportant. This will be further explored in our next step of research.

### Inequality of rural development

The inequality revealed by the SEI maps show some surprising facts, that is, the eastern Circum-Poyang Lake area should be one of the least stressed and most developed areas, but from 2010 to 2019, it has become the “least developed” area in terms of rural income and natural endowment. This is probably because of the big population lowering the income level in the agriculture-based counties, and agricultural products are usually low in price. The mountainous areas located in the south of the province should be normally the poorest but actually they are not because they benefit from plantation of economic crops such as orange (*Citrus sinensis Osb. var. brasliliensis Tanaka*) and exploitation of various mineral resources. The socio-environmental aspect has been improved in these areas. This reserves a profound thinking of decision-makers while implementing the rural revitalization measures.

## Conclusions

This study explores the spatial distribution and influencing factors of the rural residential areas in Jiangxi. As revealed by a set of spatial analysis and multiple linear regression modeling, it is evident that rural settlements and their spatial distribution are constrained by different socio-environmental factors, in particular, altitude, slope, roads, rivers and their tributaries, cultivated land resources and urban radiation. As a matter of fact, the occurrence and development of the settlements are a result of an integrated action of these factors though each factor may have a different importance in different areas. For Jiangxi province, we have obtained the following order of importance: distance to tributaries (R^2^ = 0.748), cultivated land resources (R^2^ = 0.702), effect of urban radiation (R^2^ = 0.674), distance to county-level roads (R^2^ = 0.593), elevation (R^2^ = 0.522) and slope (R^2^ = 0.520), etc. Our study makes up for the lack of geospatial research on large-scale rural settlements at regional level.

We surprisingly found that rural per capita income is negatively correlated with environmental factors such as elevation and slope and positively with cultivated land resources. The SEI developed based on this understanding may provide relevant insight on the development inequality of the rural areas in the studied province from a socioeconomic and environmental view in stead of using a single economic indicator as Gini or Engel’s Coefficient does.

With the implementation of China's first strategy “Main Functional Area Planning of Land” since 2011^63^, in particular, the strategy “Rural Revitalization” and the new policy for Construction of the Beautiful Countrysides^34^, it is possible to take advantage of these opportunities to carry out a renovation of the rural environment, for example, relocation for the scattered rural settlements, and rearrangement for the settlements in an appropriate space to ensure the safety of people's lives and property, mobilization of the urban functions to the rural areas, and so on. Our study may provide reference and target areas of priority for achieving these missions in the province, for example, areas with the SEI of < 0.4 will be recommended as target of priority for implementation of the revitalization strategy.

Although the study was conducted at regional-scale taking Jiangxi as an example, the methodology and the new index SEI can be extended to other provinces in China and even to other developing and developed countries in the world for assessing the inequality of development.
